# Protocol Improvements for Low Concentration DNA-Based Bioaerosol Sampling and Analysis

**DOI:** 10.1371/journal.pone.0141158

**Published:** 2015-11-30

**Authors:** Irvan Luhung, Yan Wu, Chun Kiat Ng, Dana Miller, Bin Cao, Victor Wei-Chung Chang

**Affiliations:** 1 SinBerBEST Program, Berkeley Education Alliance for Research in Singapore, Singapore; 2 School of Civil and Environmental Engineering, Nanyang Technological University, Singapore, Singapore; 3 Singapore Centre on Environmental Life Sciences Engineering, Nanyang Technological University, Singapore, Singapore; 4 Interdisciplinary Graduate School, Nanyang Technological University, Singapore, Singapore; Peking University, CHINA

## Abstract

**Introduction:**

As bioaerosol research attracts increasing attention, there is a need for additional efforts that focus on method development to deal with different environmental samples. Bioaerosol environmental samples typically have very low biomass concentrations in the air, which often leaves researchers with limited options in choosing the downstream analysis steps, especially when culture-independent methods are intended.

**Objectives:**

This study investigates the impacts of three important factors that can influence the performance of culture-independent DNA-based analysis in dealing with bioaerosol environmental samples engaged in this study. The factors are: 1) enhanced high temperature sonication during DNA extraction; 2) effect of sampling duration on DNA recoverability; and 3) an alternative method for concentrating composite samples. In this study, DNA extracted from samples was analysed using the Qubit fluorometer (for direct total DNA measurement) and quantitative polymerase chain reaction (qPCR).

**Results and Findings:**

The findings suggest that additional lysis from high temperature sonication is crucial: DNA yields from both high and low biomass samples increased up to 600% when the protocol included 30-min sonication at 65°C. Long air sampling duration on a filter media was shown to have a negative impact on DNA recoverability with up to 98% of DNA lost over a 20-h sampling period. Pooling DNA from separate samples during extraction was proven to be feasible with margins of error below 30%.

## Introduction

Interest in bioaerosol research has grown rapidly over the past few decades, spurred by the advent of analytical methods based on DNA. There are numerous reasons for interest in measuring bioaerosols. Some research has been motivated by potential human health effects [1,2]. While airborne microorganisms can be benign or even beneficial to humans, [3,4], bioaerosols are also known to cause infections, allergenic responses and toxic effects ranging from relatively common asthma and sick building syndrome to epidemic diseases (such as SARS) and bioweapon use (such as Anthrax) in extreme cases [5–8]. Indirect impacts of bioaerosols, such as crops infection [9], biodegradation of building materials [10,11] or how it can be transported widely by the wind, affecting life along the way [12–14], have also attracted equal attention to understand the dynamics of bioaerosols around us. Such interest to measure or identify bioaerosols in different environments, however, is seldom coupled with satisfactorily robust technical capabilities given the limitations of current culture-based and DNA-based sampling and analysis methods for filter and liquid samples. Researchers are faced with problems such as collecting enough biomass in a relatively short time (low time resolution)[15], low sampling efficiency or removing unique inhibitors from environmental samples.

The road to developing the much needed standardized methods for bioaerosol research has been challenging mostly due to major technical limitations. First, bioaerosol concentrations are naturally dilute in the environment [13,16–19]. The low concentrations of interest lead to detection limits and sensitivity problems in subsequent analysis. The second issue is the fact that bioaerosols are highly dynamic in time and space [13,20–23]. This large variability makes it difficult to establish and compare protocols from various studies.

Culture-based and microscopic methods dominated early bioaerosol research [2,20,24] and are still used today [25–28] because of their practicality and specificity. However, microscopic methods are generally labour intensive, while the culture based methods might carry a certain level of bias particularly when a broader range of microorganisms are targeted. The culture based methods also require the captured organisms to be both viable and culturable on the selected medium, which only accounts for less than 5% of total organisms in the air [18,29,30]. Furthermore, some sampling approaches may cause some stress to the targeted microorganisms and lead them, to completely or partially, lose their viability during or after collection [16,31,32].

To complement culture-based assessments, culture-independent methods have been developed. Several bioaerosol analysis protocols were built based on biological entities such as endotoxin [33,34] or glucans [35]. While these methods have merit, DNA-based analysis, which uses PCR (polymerase chain reaction) as the core technology has emerged to assume the main role [16,17,36,37] in recent bioaerosol studies. Unlike other biological entities, DNA is ubiquitous in all living things. This advantage has driven a variety of DNA-based technology, such as time-saving commercial DNA extraction kits (MOBIO, Qiagen, etc.), DNA quantification devices (Nanodrop, Qubit, qPCR), and advanced DNA sequencing technologies [38–40] to flourish with a trend towards increased technical capabilities and rapidly decreasing unit costs.

The focus of this study is to deal with challenges associated with low bioaerosol concentration from complex environmental samples, utilizing culture-independent, DNA-based analysis methods. Previous studies have commonly applied three approaches to optimize DNA yield from low biomass samples, which include: (1) extended sampling with high flowrate or long duration [16,41]; (2) improving the sample extraction process [14,42,43]; and (3) attempting to concentrate samples [8]. However, the use of these approaches is often not complemented with details on to what extent or how these efforts directly affect final results. This study aims to provide a thorough investigation of how the following three factors may improve DNA yield from bioaerosol environmental samples: (i) the effect of thermal sonication on DNA yield; (ii) the effect of long-duration filter sampling on nonviable DNA based analysis; and (iii) an alternative method to pool several low concentration samples; may improve DNA yield from bioaerosol environmental samples.

## Materials and Methods

Investigations of bioaerosol sampling, DNA extraction and analysis are the key components for this study. First, we examined the effect of additional lysis to enhance DNA extraction. Second, we investigated the impact of sampling duration on the recoverability of DNA from filters. Third, we investigated a DNA-pooling approach to overcome the analytical limitations of low DNA concentrations in environmental bioaerosol samples.

### Sampling

We tested the approaches proposed in this study on two types of environmental samples with different biomass loadings. Filter-based ambient air samples were collected to represent low biomass samples. Dust extracted from used mechanical-ventilation-system filters represents high biomass samples. The latter filters were obtained during regular air-handling unit (AHU) filter replacement in buildings of the Nanyang Technological University (NTU), Singapore. All AHU filter samples were collected with permission from NTU facilities management office and the servicing company (SMM Pte. Ltd., Singapore).

An open balcony at NTU was chosen as the location for all ambient air sampling in this study. Human contribution at that site is minimal except for brief times when researchers came to conduct sampling activity. Temperature and relative humidity (RH) measurements were routinely recorded along with filter sampling.

The time table of ambient air sampling activities can be obtained from S1 File. All samples were collected during summer 2013 by means of filtering air (without any size-cut) onto a polyethersulphone (PES) filter membrane (47mm, 0.2 μm pore size, Pall Corporation, USA) using a diaphragm vacuum pump (HCS Scientific, Singapore) with a flow rate of 16 L/min for 8 to 20 hours.

Filters from three AHUs at NTU were acquired from regular maintenance activities. These filters were in service for three months prior to acquisition. Details regarding filter collection can also be seen in S1 File. These filters are indoor secondary units, which come after the primary filter that treats only outdoor air. The secondary filter served as a filter for a mixture of both recirculated indoor air plus make-up outdoor air.

### DNA Extraction

MOBIO Power Water (PW, MOBIO Carlsbad, USA) DNA extraction kits were used for all extractions. The ambient air filters were directly put into the 5-ml bead beating tube of the PW kits to proceed with DNA extraction using flame sterilized tweezers and working under a biosafety hood to reduce potential contamination. The AHU filters were first cut into smaller pieces (1 cm × 5 cm per piece) and then directly placed in the bead beating tube for extraction.

We first conducted an experiment to determine the optimum temperature for water-bath sonication. The DNA extraction followed the original MOBIO PW protocol with an additional 30 minutes of water bath sonication and thermal incubation at different temperatures (Elmasonic, SH250EL, Germany) before the bead-beating step. The temperature settings include no-treatment (original MOBIO PW protocol), water-bath sonication only (no heating), 55°C, 60°C, 65°C, 70°C and 75°C sonication. Total extracted DNA was then directly measured by fluorometry (Qubit, Life Technologies, USA) and gene copy numbers of bacterial (16S) and fungal (18S) marker genes were estimated by qPCR (LC480, Roche Scientific, Switzerland).

We further examined the effect of sonication and heat incubation using the temperature from the previous experiment on two additional sets of environmental samples: ambient air samples (low biomass) and AHU filter samples (high biomass). The DNA extraction of one set of samples followed the original MOBIO PW protocol, while the other used additional high temperature sonication. The final DNA yields were analysed with Qubit and qPCR.

### Sampling Duration Experiment

**Fig 1:**
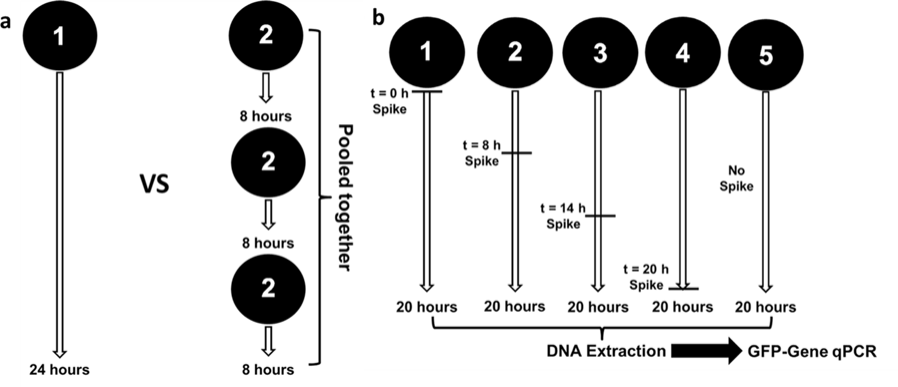
Sampling duration experiments for microorganisms collected on filters. **(a)–Comparison of 1×24 hour versus 3×8 hour parallel sampling.** Two sets of sampling trains collected air at the same time and location for 24 hours. The first set sampled the air continuously for 24 hours on a single filter, while the other set’s filter was replaced every 8 hours. DNA extracted from the three 8-h filters was then pooled and the result is compared with the first set which was extracted alone. **(b)–Testing loss of GFP-tagged bacteria during sampling**. A set of filters sampled air at the same time and location for a total duration of 20 hours. Known quanta of GFP-tagged *S*. *oneidensis* cells were spiked onto the filters at staggered timepoints, exposing them to different durations of air sampling.

Two experiments shown in Fig 1 were carried out to assess the effect of sampling duration on extracted DNA results. Fig 1A illustrates the first experiment, which employed a pair of identical sampling trains operated in parallel. With one train, the air was sampled continuously for 24 hours using a single filter. The second train also operated for 24 hours, but the filters were replaced with new ones at eight-hour intervals so that three sequential filters were collected in all. All four filters were then processed. The one 24-hour filter was extracted alone, while the DNA extracted from three 8-hour filters was pooled into a single DNA solution. Qubit measurements and qPCR were then applied to the final DNA solutions. The purpose of this investigation was to assess whether there is any DNA loss associated with long-duration filter-based sampling. If DNA degrades with extended sampling, then the yield recovered from three separate filters would be higher in sum than the single 24-h filter, since these sequential filters are each exposed to a lesser duration of air sampling.

Fig 1B illustrates another experiment, which was performed utilizing a species of Gram-negative bacteria which belongs to the Phylum *Proteobacteria*. Green-fluorescent protein (GFP)-tagged Gram-negative bacteria *Shewanella oneidensis* [44] was chosen for this experiment as this species is unlikely to present in the ambient air. Quanta of these bacteria were spiked onto filter samples at different times during ambient-air bioaerosol sampling, exposing the *Shewanella* cells to airstreams for different sampling durations. Filter 1 was spiked at 0 h (beginning of sampling), filter 2 at 8 h, filter 3 at 14 h, filter 4 at 20 h (i.e., after completion of sampling) and one set of filters was processed without any spike as the blank. Utilizing information from a preliminary experiment, we spiked the filters with an amount of cells that is comparable to the total DNA mass normally collected during 20 hours air sampling at this particular location, i.e., 10^6^ cells counts. After sampling, DNA was extracted using the same protocol described previously. A qPCR analysis was also performed using custom designed GFP-sequence primers and probes.

### DNA Concentration / Pooling Approach

**Fig 2:**
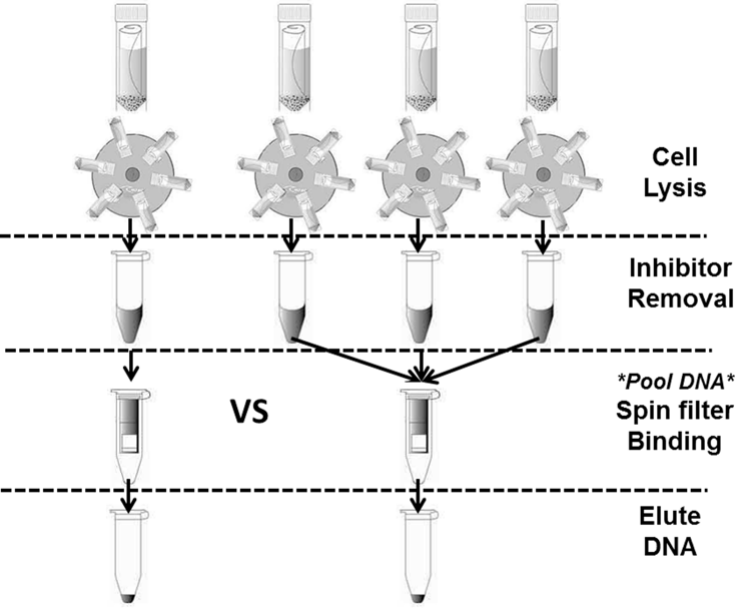
Concentration approach during extraction. The DNA from three filters is pooled during the spin-filter binding step of DNA extraction. The total DNA yield is then compared to the one filter extracted alone to investigate whether the expected 3-to-1 ratio is obtained.

A concentration approach is proposed in this study to investigate whether more biomass could be gathered from several low concentration samples without necessitating amplification either by PCR or via culturing. Fig 2 illustrates an approach to concentrate DNA in the middle of DNA extraction process.

To test the concept, four sets of AHU filters and ambient air samples, consisting of four replicates each (32 in total) were prepared and extracted in this manner. For each set of four filters, the DNA solutions from three filters were introduced onto the same spin filter after the lysis and inhibitor removal step, whereas the remaining one filter was extracted alone. The extracted DNA concentrations were used to verify whether the expected 3-to-1 ratios were obtained for both high and low biomass samples.

### DNA Measurement: Qubit and qPCR

DNA fluorometry (Qubit, Life Technologies, USA) and quantitative PCR (Roche LC 480, Basel, Switzerland) were applied to quantify and verify the quality of the extracted DNA solutions. The double stranded (ds) DNA high sensitivity kit was used for all of the Qubit assays. Qubit measures total DNA concentration in terms of ng of DNA per μL of elution liquid (ng/μL). The total mass of DNA (ng) is then calculated by multiplying the concentration with the amount of elution liquid used during extraction. For low concentration air samples, the derived DNA concentration is expressed in terms of ng of DNA per volume of air sampled through the 47-mm PES filter (ng/m^3^ of air). For the higher biomass AHU filter samples, the final total DNA concentration is expressed in terms of total extracted DNA per area of filter segmented for extraction (ng/cm^2^ of filter).

Quantitative PCR was also carried out to estimate concentrations of DNA associated with specific microbiological targets, namely bacteria, fungi and GFP. Quantitative PCR is based on the concept of DNA replication, doubling the number of targeted DNA sequences every cycle. The number of cycles needed for each sample to cross a set point is recorded as the Cp (crossing point) value. Roche LC 480 uses second derivative maximum analysis method which records the point where acceleration of the fluorescence signal is at its highest as its set point to determine the Cp value. The lower the Cp value, the higher the amount of targeted DNA sequence in the sample as fewer cycles of DNA duplication are needed to reach the set point. In addition, each Cp unit reflects a doubling of DNA abundance, approximately a doubling of mass.

Standard curves (Cp value vs. DNA mass concentration) were established for general bacteria, fungi and GFP based on Qubit measurements of extracted pure bacteria (*E*. *coli*), fungus (*Aspergillus sp*.) and GFP-tagged *Shewanella oneidensis*, which were chosen as equivalent representatives (S1 File). All the Cp values were then converted to partial DNA concentration (pg/μL) based on their respective standard curves. Similar to the Qubit result calculations, the partial DNA concentrations were finally converted to pg of DNA per m^3^ of air (pg/m^3^) for ambient air samples and to pg of DNA per area of filter (pg/cm^2^) for AHU filter samples.

Three sets of primers and probes were used for the qPCR assay. These are sequences from 16S region for general bacteria, 18S region [45] for general fungi and the GFP gene for the experiments involving *Shewanella oneidensis*. Table 1 lists the detailed sequences of the aforementioned primers and probes, each of which was synthesized by TIB Molbiol (Berlin, Germany).

**Table 1:** Probe-and-primer sets for qPCR assays.

Function	Sequence
Forward primer, 16S (bacteria)	5’–ACTCCTACGGGAGGCAG–3’ BAC338F
Reverse primer, 16S (bacteria)	5’–GACTACCAGGGTATCTAATC–3’ BAC805R
Taqman Probe, 16S (bacteria)	6FAM–TGCCAGCAGCCGCGGTAATAC–3’–BBQ BAC516F
Forward primer, 18S (fungi)	5’–GGRAAACTCACCAGGTCCAG–3’ FungiQuant-F
Reverse primer, 18S (fungi)	5’–GSWCTATCCCCAKCACGA–3’ FungiQuant-R
Taqman Probe, 18S (fungi)	6FAM–TGGTGCATGGCCGTT–3’–BBQ FungiQuant-PrbLNA
Forward primer, GFP gene	5’–ATGGAAACATTCTTGGACACAAATTG–3’ GFP-S
Reverse primer, GFP gene	5’–GTTGATAATGGTCTGCTAGTTGAACG–3’ GFP-R
Taqman Probe, GFP gene	6FAM–TCCATTCTTTTGTTTGTCTGCCATGATGT–BBQ GFP-TM

Specific to the DNA pooling performance test and the impact of sampling duration on the Gram-negative GFP-tagged bacteria test, part of the data is reported in terms of total recovered DNA (ng or pg) as the purpose of the experiments was to investigate the amount of DNA recovered regardless of concentration. To obtain these measures, the DNA concentrations measured by both Qubit and qPCR (ng/μL and pg/μL) were multiplied by the elution volume used during extraction to obtain the final extracted DNA mass for each sample (ng for Qubit and pg for qPCR).

## Results

### Sonication and Thermal Incubation

**Fig 3:**
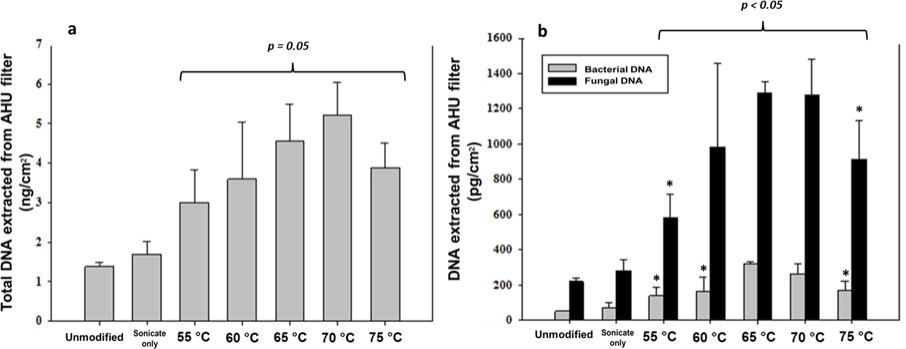
DNA measurements of AHU filter samples for seven treatment pathways of sonication at varying temperature. **(a)—Total DNA yields** (Qubit). **(b)–Results from bacterial and fungal qPCR**. *N* = 4 for each case. * denotes statistically significant difference to mean of DNA yield extracted with 65°C incubation.

The effects of sonication and thermal incubation on the final DNA yield are reported in terms of total DNA per cm^2^ area of filter or per m^3^ of air (as determined by Qubit), and as targeted DNA sequence per cm^2^ area of filter or per m^3^ of air (as determined by qPCR). Fig 3 shows that the DNA yields of the tested AHU filter samples increases gradually as the incubation temperature rises. The total DNA yield shown in Fig 3A was 1.48 ng/cm^2^ with no treatment, increasing to 4.56 ng/cm^2^ and 5.22 ng/cm^2^ with incubation treatment at 65°C and 70°C, respectively. The qPCR result on the same set of samples (Fig 3B) shows a similar trend. The bacterial DNA concentration rose from 51 pg/cm^2^ to 320 pg/cm^2^ with incubation at 65°C. The bacterial DNA yield then declined to 262 pg/cm^2^ with incubation at 70°C and 168 pg/cm^2^ with incubation at 75°C. Similarly, the highest concentration of fungal DNA (1290 pg/cm^2^) was also obtained from samples incubated at 65°C. The concentration of fungal DNA remained relatively constant for samples incubated at 70°C before decreasing to 914 pg/cm^2^ at 75°C.

Based on these results, 65°C was chosen as the incubation temperature for the remaining investigations in this study. To further evaluate the applicability of sonication and thermal incubation to a broader range of biomass concentrations, these parameters were applied to two sets of environmental samples: a set of AHU filters collected from a different building (high biomass samples) and a set of ambient air filters (low biomass samples).

**Fig 4:**
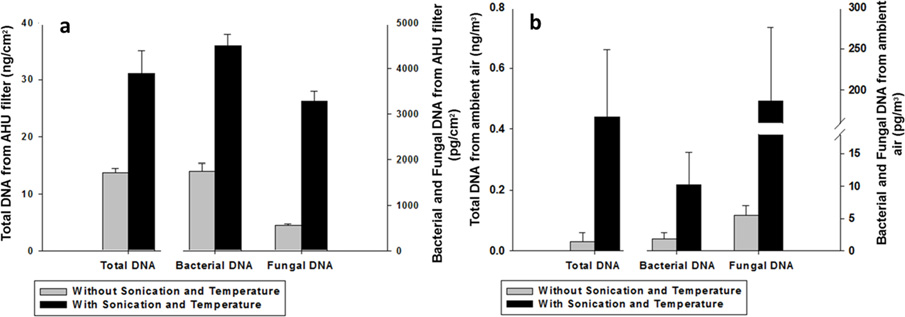
Improving DNA yield with additional heat and sonication lysis. Additional sonication and thermal lysis show improved DNA yield for **(a)—AHU filter samples** and **(b)–ambient air samples** as measured by the Qubit fluorometer for total DNA (left bar, left axis) and by qPCR for bacterial (middle bar, right axis) and fungal (right bar, right axis) DNA (*N* = 4).

The total DNA extracted from both sets of environmental samples increased substantially with the addition of sonication at 65°C, as shown in Fig 4A and 4B. For the AHU filter samples, the additional sonication and heat incubation boosts the mean of total DNA yield from 14 to 31 ng/cm^2^, more than a two-fold increase (Fig 4A, left bar). The DNA yield of the ambient air samples increases from 0.03 ng/m^3^ to 0.44 ng/m^3^ (Fig 4B, left bar), more than a ten-fold increase.

As also seen in Fig 4A and 4B (middle and right bar), the qPCR results show a similar trend to the Qubit fluorometry data, displaying a substantial jump in DNA concentration after sonication and thermal incubation for both fungi and bacteria. Fungal DNA concentrations increased from 5.5 pg/m^3^ to 187 pg/m^3^ (34×) and from 557 pg/cm^2^ to 3280 pg/cm^2^ (6×) for ambient air and AHU filter samples, respectively. Bacterial DNA concentrations went from 1750 to 4490 pg/cm^2^ (2.6×) for the AHU filters samples and from 1.9 to 10.3 pg/m^3^ (5.5×) for the ambient air samples. Thus, sonication and thermal incubation was seen to be very effective for enhancing the amount of DNA extracted from both fungi and bacteria from air. Generally, the results show that such an additional processing step might be crucial for analysing low concentration bioaerosol samples.

### Effect of Sampling Duration on DNA Yield

Two sets of experiments were conducted to investigate the effect of filter sampling on the extracted DNA yield. The first set (Fig 1A) compares the DNA yield from one filter sampled continuously for 24 hours with the yield from three separate filters sampled for 8 hours in series, the DNA from which was then combined into one concentrated sample. Similar to previous experiment, the DNA concentration of the samples are presented in terms of ng of DNA per volume of air sampled (ng/m^3^ of air) for total DNA measurement by the Qubit and for partial DNA concentration (pg/m^3^ of air) by qPCR.

**Fig 5:**
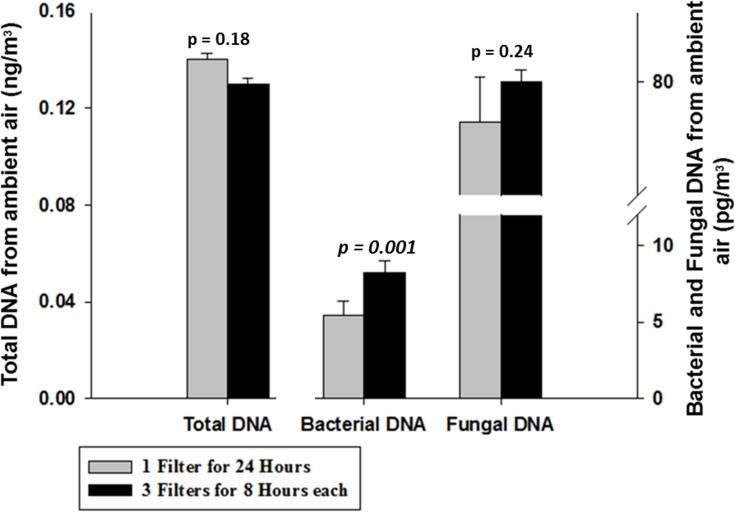
Comparison of two sampling approaches. Comparison of a sampling approach utilizing a single filter continuously sampled for 24 h (grey bar) and a combined series of three filters, each operated for 8 h (black bar) expressed in terms of total DNA (left bar, left axis) measured by Qubit and in terms of bacterial (middle bar, right axis) and fungal (right bar, right axis) DNA measured by qPCR (*N* = 3).

Fig 5 (left bar) shows that—based on DNA fluorometry—there is no distinct difference in total DNA yield between the 1×24 hour samples (0.14 ng/m^3^ of air) and the 3×8 hour samples (0.13 ng/m^3^ of air) (Paired t-test, p value = 0.18). This result led us to further analyze the DNA by performing fungal and bacterial qPCR analysis to investigate whether different yield can be seen for different microbiological targets using these two sampling approaches.

Bacterial and fungal DNA analysis (Fig 5, middle and right bar) indicates that there is no significant difference in fungal DNA concentration with 77 pg/m^3^ and 80 pg/m^3^ for the 1×24 and 3×8 hour samples, respectively (Paired t-test, p value = 0.32). However, there is a clear gap between the two sampling techniques for bacterial DNA (Paired t-test, p value = 0.001). The 1×24 hour samples yield 5.4 pg/m^3^ of air, whereas the 3×8 hour samples yield 8.2 pg/m^3^, an increase of more than 50%. This result suggests that bacterial DNA may be more sensitive to sampling duration than fungal DNA. If so, then shorter sampling time might be particularly important for preserving bacterial DNA. Fungal DNA appears insensitive to sampling duration on filter media.

The second experiment on the influence of sampling duration was performed by spiking the same amount of GFP-tagged *S*. *oneidensis* cells on four different filters at different times. These parallel samplings were conducted simultaneously at same location for a period of 20 hours while the DNA spiking was applied at t = 0, 8, 14, and 20 h, as illustrated in Fig 1B. As the main goal of this experiment was to understand the effect of long sampling duration on cells of a known bacterial species, the result in Table 2 is only presented in terms of total DNA mass estimated (pg) by GFP-specific qPCR.

**Table 2:** qPCR results from a series of 20-h samples illustrating the decay of GFP-tagged *Shewanella oneidensis* cells.

Sample treatment ^a^	No. of replicates	Estimated mass of GFP DNA recovered (ng) ^b^	Exposure to air sampling (hours)	% of DNA retained (relative to t = 0)
No spike	3	0.02 ± 0.001	-	-
Spike at *t* = 0	6	0.13 ± 0.02	20	1.3%
Spike at *t* = 8 h	3	0.73 ± 0.01	12	7.1%
Spike at *t* = 14 h	3	1.13 ± 0.02	6	11%
Spike at *t* = 20 h	6	10.2 ± 0.3	0	100%

^a^ Time (*t*) during the 20-h sampling period when a spike of *S*. *oneidensis* cells was applied to the filter.

^b^ Mean ± standard deviation reading from qPCR analysis using GFP probe and primers

Table 2 indicates that there is a diminishing trend of recoverable DNA with longer sampling duration. Up to 98% of DNA loss is shown upon exposure of the spiked filter to 20 hours of subsequent sampling. For the filters spiked at the end of air sampling, 10.2 ng of GFP gene DNA was recovered, whereas only 0.13 ng (1.3%) of GFP gene DNA was recovered from filters spiked at t = 0. The DNA recovered from the filters spiked at 8 h and 14 h were intermediate between these two limiting values: 0.73 ng (7.1%) and 1.13 ng (11%) respectively. This result suggests that much of the biomass collected at the beginning of an extended sampling period could be lost throughout subsequent sampling, especially for species more susceptible to stress from air sampling.

### Mid-Extraction Pooling or Concentration Approach

The performance of DNA pooling during the extraction step was analysed by means of Qubit measurements and qPCR in terms of total DNA mass extracted (ng or pg). The result compares the DNA yield of one filter and the yield of three replicate filters combined.

The DNA is first extracted to a certain extent and combined into one concentrated sample during the spin filter binding step of the MOBIO PW protocol (Fig 2). Table 3 displays the ratios of biomass extracted from one and three filters which range from 1:2.5 to 1:3.9. These ratios are close to the theoretical value for lossless concentration of 1:3.

**Table 3:** qPCR cycle threshold value data for mid-concentration approach considering both 16S bacterial and 18S fungal probe-and-primer sets.

**Ambient Air Samples**	**Mean Total DNA** ^**a**^ **(ng)**	**Mean DNA Concentration** ^**b**^ **(ng/m** ^**3**^ **)**	**Mean Bacterial DNA** ^**a**^ **(pg)**	**Mean DNA Concentration** ^**b**^ **(pg/m** ^**3**^ **)**	**Mean Fungal DNA** ^**a**^ **(pg)**	**Mean DNA Concentration** ^**b**^ **(pg/m** ^**3**^ **)**
1 Filter	3.1 ± 0.9	0.3	167 ± 37	17.4	1481 ± 142	154
3 Filters	11.9 ± 1.9	0.4	414 ± 114	14.4	3780 ± 440	131
Ratio	1: 3.9		1: 2.5		1: 2.6	
Concentration Deviation (%)	28%		21%		18%	
**AHU Filter Samples**	**Mean Total DNA (ng)**	**Mean DNA Concentration (ng/cm** ^**2**^ **)**	**Mean Bacterial DNA (pg)**	**Mean DNA Concentration (pg/cm** ^**2**^ **)**	**Mean Fungal DNA (pg)**	**Mean DNA Concentration (pg/cm** ^**2**^ **)**
1 Filter	44 ± 6	4.4	570 ± 46	57	2970 ± 410	297
3 Filters	148 ± 12	4.9	1798 ± 124	60	10100 ± 1420	338
Ratio	1: 3.4		1: 3.2		1: 3.4	
Concentration Deviation (%)	12%		5%		14%	

^a^ Mean ± standard deviation for *N* = 4 samples in each case

^b^ Concentrations calculated from total DNA extracted divided by total amount of air sampled through one and three filters (for ambient air samples) or total area of AHU filter extracted (for AHU filter samples).

The calculated concentrations per unit volume of air sampled (ng/m^3^) and per unit area of AHU filters (ng/cm^2^) in Table 3 are expected to be the same (between one and three filters) as these comparisons were made from replicates of the same samples. The difference of concentrations calculated from one and three filters are relatively small with a margin of error of ≤ 28% (using the results from one filter as the reference). The high concentration AHU filter samples display smaller deviations, ranging from 5 to 14%. Conversely, the low concentration ambient air samples have higher deviation ranging from 17 to 28%.

## Discussion

### DNA Extraction: Cell Lysis

The extraction step plays a big role in the effectiveness of DNA-based analysis of environmental samples. As DNA-related technology grows, a variety of extraction kits are continuously improving the efficiency in both DNA yield and extraction time. The MOBIO Power Water (PW) kit was selected in this study mainly due to its frequent use in previous bioaerosol studies [14,42], as well as compatibility with the size of our filter samples. The PW kit uses 5 ml sample tube which allows filter samples to be directly placed inside the tubes (47 mm PES and the 5 cm^2^ cut AHU filters) without having to scrape, grind, vacuum or dissolve the filters before proceeding with extraction steps. This feature minimizes extraction time and biomass loss. Choosing the appropriate kits design and size is important. Other bioaerosol studies have chosen other extraction kits such as the MOBIO Power Max Soil kit for processing larger sample volumes in 50 ml tubes [46] or MOBIO Power Soil for more rigorous inhibitor removal when smaller starting tubes (2 ml) can be used [47].

DNA extraction consists of three main steps: 1) cell lysis to expose the intracellular material; 2) isolation of DNA from contaminants; and 3) final elution. For environmental samples, the challenge in DNA extraction often comes from the lysis and isolation steps. Environmental samples start with a mixture of species with different characteristics, which may require different approaches to effectively lyse all the cells. Moreover, if inhibitors such as humic acid from floor dust or particulate matter suspended in the air remains in the final DNA solution, the integrity of subsequent analysis steps, such as qPCR, can be compromised.

As a quality control measure, a dilution series was performed on a separate set of DNA samples extracted from AHU filter and PES ambient air filter to verify potential inhibitor effects. The results suggested no lowered inhibition from the diluted samples. Furthermore, most of our qPCR results correspond well with the Qubit measurements and all of our final DNA samples are clear in colour. Given the fact that some of the samples had high dust contents (e.g. the extracts from AHU filters), it seems the MOBIO PW kit was able to effectively handle the inhibitors in all environmental samples collected in this study.

Final DNA yield of an extraction is often related to its lysis. The complex matrix of bioaerosol samples has forced several studies to apply some improvements to their lysis protocols, such as sonication [14], thermal incubation [42], and chemical addition [17,43]. Three commonly used cell lysis methods are physical disruption, chemical and enzymatic lysis. This study focused on the impact of a combination of sonication and thermal incubation (physical disruption) because this approach, combined with the physical bead beating in the MOBIO protocol, is known to be effective in lysing cells with tough cell wall/membrane (spores, Gram-positive bacteria) and small bacteria cells[48]. In addition, it is relatively more flexible to be applied to almost all DNA extraction protocols. The impact of chemical and enzymatic lysis on various forms of bioaerosol samples, however, also warrants future investigations.

Choosing the optimum temperature for high temperature sonication can be challenging when applied to environmental samples with unknown quantity and composition of biomass. Ideally, one wants the temperature to be high enough to help lyse the cells, but also not too high to avoid DNA denaturation or lysis buffer compatibility issues. Some studies have chosen to incubate their samples at 60°C[49] or 65°C [14,42] without elaborating the underlying reasons for the choice. The results from Fig 3A and 3B agree that the highest DNA yields came from filter samples incubated at either 65°C or 70°C. Incubation at 65°C over 70°C was chosen for the rest of the study as it preserves more bacterial DNA (Fig 3B) and displays more consistency among the replicates (Fig 3A and 3B).

In order to verify the validity of this temperature choice, ANOVA single factor followed by Tukey honest significant difference (HSD) analysis for pairwise comparisons was performed comparing results from samples incubated at 65°C to the other temperatures (65°C to 55°C, 60°C, 70°C, and 75°C) for total, fungal and bacterial DNA. The means of total DNA for all 5 temperatures showed no significant difference from that of 65°C (p-value > 0.05). However, the analysis results of fungal and bacterial DNA provided further insights. For fungal DNA, only comparisons of 65°C to 60°C and 65°C to 70°C have p-values > 0.05. As for bacterial DNA, only comparison between 65°C and 70°C results in p-value > 0.05. The results may explain why different studies mentioned above incubated their samples at different temperatures to obtain the required DNA yield. It is likely that in the complex matrix of an environmental sample, different target biomass would behave differently under varying temperatures. Therefore, depending on the purpose of a study, various incubation temperatures may be considered. For example, when only rough estimation of total DNA via Qubit is required, any temperature from 55°C to 75°C may all produce comparable results. If the focus is on fungal DNA, temperatures from 60°C to 70°C may be chosen. Lastly, when bacterial DNA is targeted, temperatures at 65°C or 70°C could be the options as they have equally high yields.

In the present study, our focus is to preserve as much DNA as possible for further analysis of all three DNA types. The statistical analysis above shows that there are only two temperatures (65°C and 70°C) that are within the range of temperatures that produces the highest yield for all DNA types (total, bacterial and fungal). Between these two temperatures, the lower one (65°C) is chosen as the final incubation temperature because in addition to the higher consistency and better preservation of bacterial DNA, lower temperature is also preferred to save energy and further reduce the chance of DNA denaturation during processing.

During sonication, the additional force provided by heating [50] and cavitation [51] help disrupt the cell walls and subsequently release more intracellular matter, including DNA. The impact of sonication alone on DNA yield can be seen from the difference between no treatment and sonication only bars in Fig 3A and 3B. The total DNA yield goes up by 23% (Fig 3A) with additional sonication alone while bacterial and fungal DNA yield went up by 43% and 28%, respectively (Fig 3B). The effect of heat incubation is seen to dominate with up to 600% increase in DNA yield as the temperature is increased. The influences of the two approaches appear to be interrelated, with sonication playing a supporting role. It is also worthwhile to note that sonication itself dissipates heat in the water. We’ve observed that when the instrument is operated in sonication-only mode, water temperature rises to 35–40°C within the first 5 minutes and then stays in that range for the remainder of the 30-min process.

We would like to also highlight that there are indeed limitations associated with studies utilizing environmental samples. It can be observed from the results that the impact of one improvement approach (i.e. thermal sonication) varies on different target biomass. Compared to the typical controlled experiments, the complex environmental matrix makes it very difficult to estimate the absolute extraction efficiency from environmental samples. The absolute extraction efficiency can only be calculated when artificial samples with a known starting amount (spiking a clean/PM-loaded filter with a known amount of cells or using nebulizer to mimic bioaerosol sampling) are used. These efficiencies, however, are more relevant for studies targeting a specific species or a small group of species as they only correspond to the chosen cells that are spiked, which in most cases, does not represent actual environmental conditions. We believe it is beneficial to also report the improvements of an optimization approach (physical disruptions, enzyme, etc.) tested on environmental samples to complement the more readily available studies with known samples in the literature.

We further tested the improved protocol with 65°C sonication on two sets of environmental samples with significantly different biomass concentrations. The results displayed in Fig 4A and 4B show that the total DNA yield from both ambient air and AHU filter samples increased significantly with the addition of a 65°C sonication step. Note that two of four replicates for total DNA from ambient air samples without sonication (Fig 4B) were below the detection limit of the Qubit. This result highlights the importance of sonication and heat incubation in DNA extraction because it permits the direct use of DNA fluorometry to quantify total DNA mass for samples that would otherwise be undetectable. DNA fluorometry provides total DNA quantification in a relatively short time, which is an attractive feature in indoor environmental assessment. Total DNA measurement is generally not available in PCR-based techniques as their analysis is limited to the chosen primers. While DNA fluorometry (i.e. Qubit) is considered more accurate than light-absorbance-based methods, such as nanodrop [52], there are only limited reports of direct quantification of DNA in air samples using this method. This is likely due to its relatively higher detection limit (typically 0.0005 ng DNA/μL) compared to PCR-based instruments (capable of detecting DNA concentration up to 10 times lower than that of Qubit with enough duplication cycles).

As shown in Fig 4A and 4B, large deficits of biomass for both bacterial and fungal DNA in both ambient air and AHU filter samples suggests that the original MOBIO protocol (with bead beating only) does not effectively lyse all the cells in environmental samples such as these. One can speculate that the higher DNA yield from additional lysis originates from cells with thicker walls, such as fungal spores or Gram-positive bacteria, which might not be effectively disrupted by means of only bead beating.

The results of this study indicate that it would be worthwhile to be attentive to lysis as a critical processing step for environmental bioaerosol sample analysis. The total DNA measurements of ambient air samples extracted with the unimproved protocol shows that one may miss detection with inadequate lysis. The influence of an improvement approach can also vary among different types of environmental samples (in this case, ambient air filters and AHU filters). More attention towards method development would be beneficial for future bioaerosol studies, including systematic cross-comparison of improvement techniques under a range of environmental sampling conditions.

### Effect of Sampling Duration on DNA Yield

To better understand how the sampling duration affects the DNA yield from the filter sample, we have utilized an open (no size-cut) filter-based sampling protocol for its relatively higher collection efficiency (as compared to liquid impinger) [30,53], flexibility (in choosing sample flowrate and duration)[18] and cost. The naturally low bioaerosol concentrations in ambient air often lead to issues of inadequate measurement sensitivity. Therefore, to collect enough biomass, long sampling durations are sometimes inevitable for non-viable based analysis. Many studies have utilized relatively long sampling times such as eight hours [42], 20+ hours [54], or even several days for collection on individual filters [16,41].

Concerns about time-dependent decreases of the viability of captured cells during air sampling have been raised over the years. Depending on the sampling medium, microorganisms may partially or completely lose their viability owing to stress from different parameters such as shear-stress induced by air flow, osmotic pressure or lack of moisture [16,26]. Of course, DNA-based analyses do not rely on viability as the DNA can be extracted and analysed regardless of whether the captured microorganisms are viable or culturable. Nevertheless, the general concern remains: do extended sampling periods lead to degradation of previously collected DNA? The purpose of our investigation on this point was to further assess whether long sampling duration has a significant adverse impact on DNA analysis yield.

The total DNA measurement in Fig 5 (left bar) suggests that culture-independent DNA based analysis is not impeded by long sampling duration on a filter media. The qPCR result (Fig 5, middle and right bar), however, reveals that although there is no notable difference on the fungal DNA, 50% more bacterial DNA was preserved in the DNA solution extracted from the combined 3×8-h filters. As the amount of bacterial DNA is only a small portion of total DNA, which also includes fungal and other non-microbial DNA, the 50% reduction in bacterial DNA does not generate a notable change in total DNA. However, this result does suggest that some more vulnerable species, such as Gram-negative bacteria, may be prone to DNA loss from long duration sampling with filters.

The second experiment, illustrated in Fig 1B, was conducted to confirm the above hypothesis. There are two major reasons for selecting *Shewanella oneidensis* [44] for this experiment. First, this species is embodied as Gram-negative bacteria, which are relatively less-protected from air sampling due to their thinner cell membrane. We suspect that if DNA degradation were to happen during air sampling, then less protected cells would be more vulnerable. The second reason is because this GFP-tagged species is rarely found in ambient air. The ability to control the timing of the presence of this species on air filters through the spiking process permits us to isolate the impact of air sampling on DNA recoverability.

The qPCR result reported in Table 2 indicates a progressive degradation of DNA recoverability for this specific species in relation to sampling duration. This occurrence leads us to believe that without properly testing the compatibility of our target species and our chosen sampling protocol, the DNA of the cells captured at the beginning of sampling may be falsely undetected in the subsequent analysis steps. The degree of DNA recoverability loss is likely to be species dependent, as the previous results (Fig 5, middle and right bar) show that fungal DNA is less sensitive to air sampling on filter media than bacterial DNA.

The two experiments indicate that the duration of filter-based air sampling might have some effects on the recoverability of DNA. Depending on the chosen sampling medium and the characteristics of the targeted species, it would be beneficial to have a preliminary investigation to assess how durable is the biomass DNA during sampling. For instance, in the case where certain Gram-negative bacteria or other more vulnerable species are targeted, shorter sampling duration would be recommended to preserve more DNA. Conversely, in the case when tougher species like fungal spores or Gram-positive bacteria are targeted, longer-term sampling can be utilized as the DNA collected from these species is less likely to degrade from sampling stress. In addition to sampling duration, storage duration may also play a part in DNA preservation. It is recommended to extract the DNA as early as possible after sampling since some DNA on the filter may continue to degrade during storage [48].

### Mid-Extraction Pooling or Concentration Approach

Various efforts to concentrate bioaerosol samples have been reported in previous studies to circumvent detection limit issues. For example, Boreson et al. [8] concentrated 5 mL of liquid from a Biosampler by filtering the collected liquid media onto a section of filter membrane before proceeding with extraction. As commercial DNA extraction kits continue to improve, it is now possible to combine DNA recovered from separate samples in the middle of extraction steps, which saves time and, in some cases, reagents. In the case of MOBIO PW kit, the DNA can be pooled by means of introducing extraction liquid (after PW3 addition) from different samples on to the same spin filter.

One concern that arises for this concentration approach is the DNA binding capacity of the spin filter itself. According to MOBIO, the PW kit spin filter can bind up to 20000 ng of DNA, which is ample for the level of biomass processed in this study (up to 300–400 ng of DNA from 30 cm^2^ of AHU filters following 3 months of in-use service).

The ratios resulted in Table 3 are all relatively close to 3:1, confirming the suitability of this pooling approach to the range of biomass concentration engaged in this study. As discussed previously, it is sometimes necessary to combine several parallel filters into one concentrated sample to overcome instrument detection limits owing to the low airborne DNA concentration. The approach of combining DNA from three samples onto one spin filter has a margin of error below 30% for the experiments reported here. We consider this outcome to be satisfactory because these deviations are not only caused by the pooling method, but also would result from natural variability of the replicate environmental samples.

In addition to the reliability, the suggested DNA pooling method is generally applicable to all extraction kits with similar spin filters. It is not limited to bioaerosol samples. Different concentration approaches generally help gather enough biomass with shorter sampling duration, which could be beneficial to DNA recoverability. Furthermore, this concentration approach allows more flexibility in designing a suitable sampling plan, because other concerns such as noise, sampler durability or site availability often arise when intense sampling activity is needed.

## Conclusions

Collecting enough biomass during for desired analysis methods a short time period is a well-known challenge for the typically low concentrations of bioaerosol levels found in indoor and outdoor air. This study has shown that one can improve DNA recoverability and yield in bioaerosol samples by fine-tuning several parameters such as applying more rigorous cell lysis during DNA extraction or pooling composite samples to obtain enough biomass.

In the cases reported here, outdoor air and building AHU filter samples were analyzed using DNA-based measurement methods. Adding enhanced lysis by means of high temperature water bath sonication was proven to be effective with up to 600% increase in DNA yield at the optimum temperature of 65°C. We found that for some more vulnerable species, up to 98% of the captured species’ DNA could degrade over the course of 20 hours of filter sampling. Combining DNA from separate samples during the spin filter binding step of the DNA extraction protocol showed promising results with a margin of error below 30%.

We examined the impact of the aforementioned parameters using a commercially available DNA extraction kit, which generally demonstrates good capacity for urban environmental samples. The chosen kit seems to be able to effectively deal with inhibitors of both high biomass (AHU filters used for three months) and low biomass (PES filters applied to sample ambient air for 8–20 h) samples. Incorporating the proposed modifications saved both time and reagents in addition to achieving improvements in DNA yield.

Dealing with highly variable bioaerosol environmental samples, researchers are often faced with uncertainty on which approaches may best improve their analytical methods. Therefore, we believe that it is beneficial to have more method development-based bioaerosol studies in the future that assess how efficient different improvement techniques (e.g., enhanced chemical or enzyme lysis) are in dealing with other forms of environmental samples (liquid impinger samples, dust-wipe samples). More such efforts will help reach the goal of more standardized analysis methods for studies of aerosol microbiology.

## Supporting Information

S1 File Sampling Schedule and Standard Curves. Sampling schedules information and standard curves for bacteria, fungus and custom-GFP for qPCR Analysis.
(XLSX)
